# Chemical Fingerprinting and Quantification of Chinese Cinnamomi Cortex by Ultra High Performance Liquid Chromatography Coupled with Chemometrics Methods

**DOI:** 10.3390/molecules23092214

**Published:** 2018-08-31

**Authors:** Ninghui Ma, Yue Ding, Yong Zhang, Tong Zhang, Yaxiong Yi, Bing Wang

**Affiliations:** 1 School of Pharmacy, Shanghai University of Traditional Chinese Medicine, Shanghai 201203, China; mnh2029@163.com (N.M.); yyxiongpy@outlook.com (Y.Y.); 2 Experiment Center for Teaching and Learning, Shanghai University of Traditional Chinese Medicine, Shanghai 201203, China; dingyue-2001@hotmail.com (Y.D.); annabel_cn@163.com (B.W.)

**Keywords:** ultra-high performance liquid chromatography, Cinnamomi cortex, quantification, Principal component analysis, hierarchical clustering analysis

## Abstract

To rapidly clarify and quantify the chemical profiling of Cinnamomi cortex a reliable and feasible strategy of chromatographic fingerprinting with a suite of chemometrics methods was developed and validated by ultra-high performance liquid chromatography coupled with diode array detection. Furthermore, to identify more meaningful chemical markers, the chemometrics methods including hierarchical cluster analysis (HCA), principal component analysis (PCA) and similarity, which all generate quality evaluations and correlation classifications of Cinnamomi cortex, were used to improve the Cinnamomi cortex quality control standards. A total of 12 characteristic peaks were confirmed, seven of which were identified by comparing their retention times, UV and MS spectra with authentic compounds. Moreover, 11 analytes were accurately determined, as a complementary quantification method of chromatographic fingerprinting. For quantitative analyses, selective detection was performed at 254, 280 and 340 nm. The tested samples were separated and determined using UPLC and a series of methodologies including linearity, precision, accuracy, limit of detection and quantification and extraction recoveries were validated. Meanwhile the method bias for all the analytes did not exceed 5%. A total of 42 samples were acquired in China and analyzed. The results demonstrated that chromatographic fingerprinting in combination with chemometrics methods provides a promising and practical method to more effectively and comprehensively control the quality of Cinnamomi cortex from various sources, which would be a useful reference for the development and further study of Cinnamomi cortex and related formulations.

## 1. Introduction 

For thousands of years, Traditional Chinese Medicines (TCMs) have been widely used to treat different diseases in China. Meanwhile, quality control practices, as one of the core issues of TCM experimental research, is now increasingly gaining popularity as a mandatory requirement, especially for international trade of food and pharmaceuticals, and it should be well documented and characterized [1,2]. Due to the complex chemistry of TCMs and the lack of appropriate references, the methods for quality control of TCM have suffered from limitations [3]. Under the condition of unknown chemical composition, chromatographic fingerprinting is considered to be an effective method for the quality control of TCMs, reflecting the group characteristics of TCMs. As compared with other methods, chromatographic fingerprinting can describe the general characteristics of a TCM, which is appropriate for a comprehensive evaluation [4] and is widely accepted as one of the most effective and promising approaches to the quality control of TCMs [5,6]. Therefore, chromatographic fingerprinting for quality control methods has been gaining more and more attention recently [7].

In China, the areas producing Cinnamomi cortex (CC) are mainly in Guangdong Province and Guangxi Province. CC is a well-known crude drug that is frequently used in Chinese pharmaceuticals, derived from the dried bark from *Cinnamonmum cassia* according to the 2015 edition of the Chinese Pharmacopoeia. CC is an edible traditional Chinese medicine, widely used in the spice and pharmaceutical industries [8]. In addition, CC is also acknowledged as a valid herbal medicine with anti-inflammatory [9], anti-oxidant [10,11,12,13], and anti-diabetic pharmacological activities [14]. According to the Pharmacopoeia of the People’s Republic of China, *Cinnamomum cassia*, as the sole source of CC, is documented, while its adulterants from the other species, such as *Cinnamomum burmanni* and *Cinnamomum tamala*, are still being applied as CC for medicine in some areas of China [15,16]. Even if they are the same genus as *Cinnamomum cassia* Presl, they still have different therapeutic effects. Furthermore, concentration ranges of various ingredients spanning several orders for magnitude were also mentioned for a single herb [3]. Therefore, a reasonable and efficient method for the quality control of CC is urgently needed, especially for its therapeutic efficacy and safety.

Due to the pharmacological activities and therapeutic efficacy of cinnamaldehyde and cinnamic acid in CC, they were usually regarded as effective chemical markers to evaluate the quality of CC with TLC, GC-MS, or HPLC. In general, the determination of CC is usually only conducted with only few bioactive components, such as the aforementioned cinnamaldehyde [17] and cinnamic acid [18], so it is very necessary to establish a more effective analytical method for the quality assessment of CC. LC-MS is widely accepted as an effective method of the identification of TCM samples, and it is a significant advantage for chromatography separation, especially in combination with UPLC-DAD [19]. LC-MS/MS, a powerful tool is utilized to simultaneously identify complicated compound mixtures in herbal medicines [20]. Although there were some papers associated with CC multicomponent determination or chromatographic fingerprinting by various analytical techniques, such as MS, GC, HPLC [21,22], very few reports were found on quality assessment of CC with a systematic analytical method based on multivariate chemometrics techniques. 

**Figure 1:**
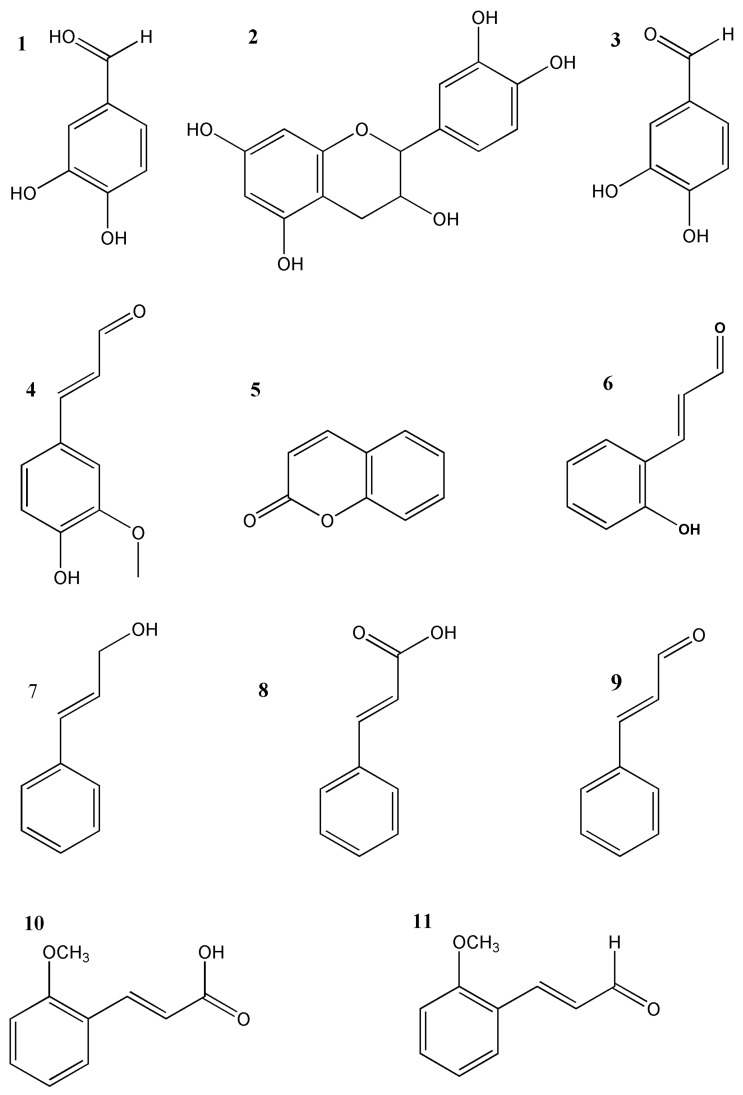
Chemical structures of 11 constituents in CC.

In the present study, a more effective strategy of chromatographic fingerprinting in combination with a suite of chemometrics methods was established for the quality control of CC. Three phenolic compounds, namely protocatechuate (**1**), L-epicatechin (**2**) and protocatechualdehyde (**3**), and eight phenylpropanoid compounds, namely coniferaldehyde (**4**), coumarin (**5**), 2-hydroxycinnamaldehyde (**6**), cinnamyl alcohol (**7**), cinnamic acid (**8**), cinnamaldehyde (**9**), 2-methoxycinnamic acid (**10**) and 2-methoxycinnamaldehyde (**11**) were used as markers (Figure 1). Chromatographic fingerprinting was established and validated by a total of 42 samples from genuine producing regions (Guangdong Province and Guangxi Province) and Chinese herbal medicine markets (Bozhou and Yulin). Chemometrics methods including similarity evaluation for chromatographic fingerprinting of TCM, principal component analysis (PCA), hierarchical clustering analysis (HCA) were further applied to analyze the data of chromatographic fingerprinting and to produce a visual evaluation for the similarities and differences of materials from different sources.

## 2. Results and Discussion

### 2.1. Optimum Conditions for HPLC-DAD Analysis

**Figure 2:**
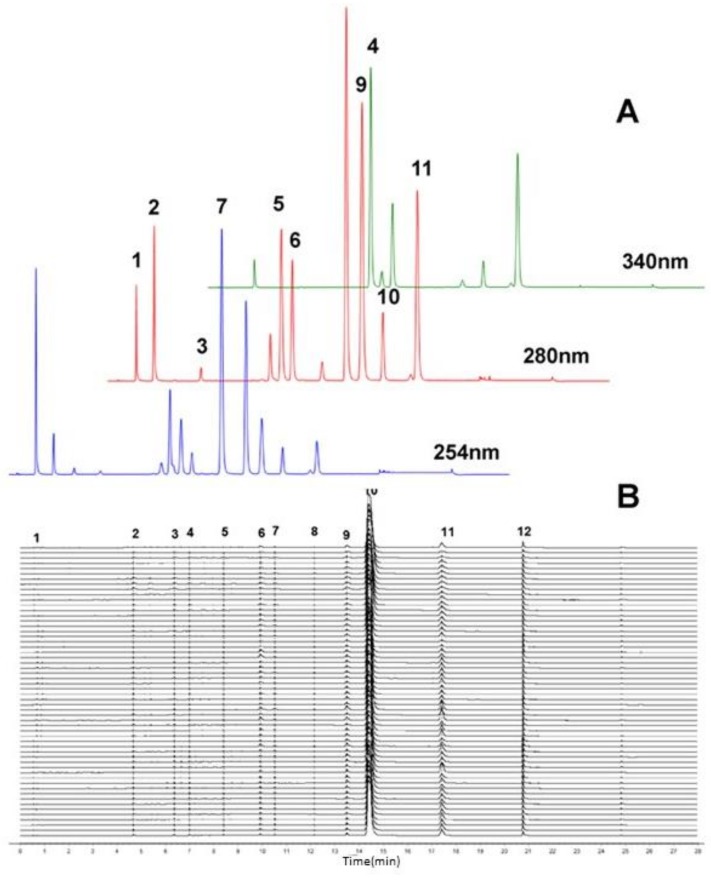
Multiple chromatograms (**A**) of 11 constituents of CC: 1 Pro; 2 Le; 3 Prod; 4 Con; 5 Cou; 6 Hc; 7 Cal; 8 Cac; 9 Cin; 10 Mca and 11 Mc; Similarity analysis (**B**) of the 11 constituents of 42 batches.

Various HPLC parameters such as detection wavelength, mobile phase and column temperature were optimized to achieve a satisfactory separation and a good shape of the chemical compound peaks in CC. After several different HPLC parameters were tried, a set of optimum HPLC parameters were reached, which consisted of a column temperature of 40 °C and a detection wavelength of 280 nm for protocatechuate (Pro), L-epicatechin (Le) and protocatechualdehyde (Prod), coumarin (Cou), 2-hydroxycinnamaldehyde (Hc), cinnamic acid (Cad), cinnamaldehyde (Cin), 2-methoxycinnamic acid (Mca) and 2-methoxycinnamaldehyde (Mc); 254 nm for cinnamyl alcohol (Cal); 340 nm for coniferaldehyde (Con). By taking the acetonitrile-water mobile and methanol-water phase systems into consideration, it was found that the 0.025% formic acid acetonitrile-water system gave better resolution and better peak shapes than the methanol-water system. The chromatograms of the 11 compounds obtained under the optimized chromatographic conditions are presented in Figure 2A.

### 2.2. Similarity Evaluation

The similarity evaluation of the chromatographic fingerprints was performed to assess geographical differences, where original data of samples were analyzed using the original correlative coefficient data. To further evaluate the quality of CC, the optimized UPLC-DAD method was used for analysis of 42 batches of CC from different sources. The Similarity Evaluation System software (Version 2004A, the State Food and Drug Administration of China, Beijing, China) for the chromatographic fingerprinting of TCMs (version 2.0) was applied for fingerprinting these chromatograms (Figure 2B).

The reference characteristic fingerprint of CC was constructed from the average chromatograms of 42 CC batches, which subsequently generated the similarity values with other CC chromatograms. Twelve common peaks were assigned as characteristic peaks with good resolution, which were found in all 42 batches of samples, seven of which were determined in the HPLC fingerprint chromatograms. Peak **9**, with good shape, a stable area and moderate retention time in the HPLC fingerprint chromatograms, was assigned as the reference peak. The similarity of each chromatogram to the reference characteristic fingerprint was investigated. The similarities of the 42 samples were all over 0.99, which indicated that there is no obvious difference in the basic chemical composition. The results indicated some similarities as well as marked differences. Twelve characteristic peaks were common in all samples. The fingerprints could give a comprehensive evaluation of the quality of CC as a whole, and make up for the limitation of the determination of content. The similarity results provide a useful reference for the relationship among chromatographic fingerprinting of materials from different sources. 

### 2.3. Principal Component Analysis (PCA)

PCA is famous for its broad data matrices, including masses of variables in the chromatographic fingerprints. It has been reported that PCA is an efficient tool, whereby several principal components were extracted to verify the most possible variability based on the multivariate variation data [23,24]. Meanwhile, PCA score plots indicated a visual variation among groups. The areas of 12 characteristic peaks of 42 batches of CC samples were selected as the clustering variable.

**Figure 3:**
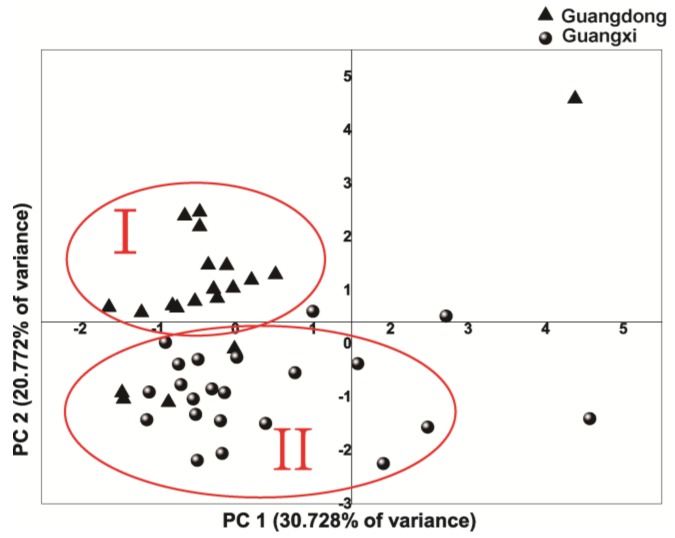
Score plot of principal analysis (PC1-PC2) of 42 bark samples of CC.

The correlations of the contents of major chemical constituents were illustrated by PCA. The data of 12 characteristic peaks were selected to standardize for feature extraction of principal component (PCs). In the score plot, on the basis of eigenvalues>1, four principal components accounting for 75.9% of the total variance were considered significant. A macroscopic classification appeared in the score plot of the first two principal components (Figure 3), with which the samples could be discriminated according to different regions. From the scatter points, the samples could be classified into two obvious main groups (Cluster I: Guangdong and Cluster II: Guangxi Province), which were distinguished as groups depending on different provinces. This showed the geographical relationships and distribution patterns among CC. It was worth mentioning that S12, S13, S14 from Cluster I were divided into Cluster II, which demonstrated that a closer geographical relationship was found with Guangxi Province rather than Guangdong Province. The sample records from the edges of Guangdong Province and Guangxi Province also confirm this point. Moreover, compared with those of Guangdong Province, the samples from Guangxi Province have a larger scattered region, which suggested that the samples from Guangxi Province had a greater quality variation than those of Guangdong Province. However, a large drift was found in a few samples, which indicated a remarkable difference in composition or content in comparison with other samples. The results indicated different origins were the main reason of variations of principal component constitute and amount. Moreover, these factors, including climate, soil type, harvesting time, storage condition and harvesting and processing should also be considered. Geographical differences were very revealing in the results of classification. A simplified form of the data was obtained for the quality assurance of CC by evaluating the variation and similarity between the samples. 

### 2.4. Hierarchical Clustering Analysis (HCA)

**Figure 4:**
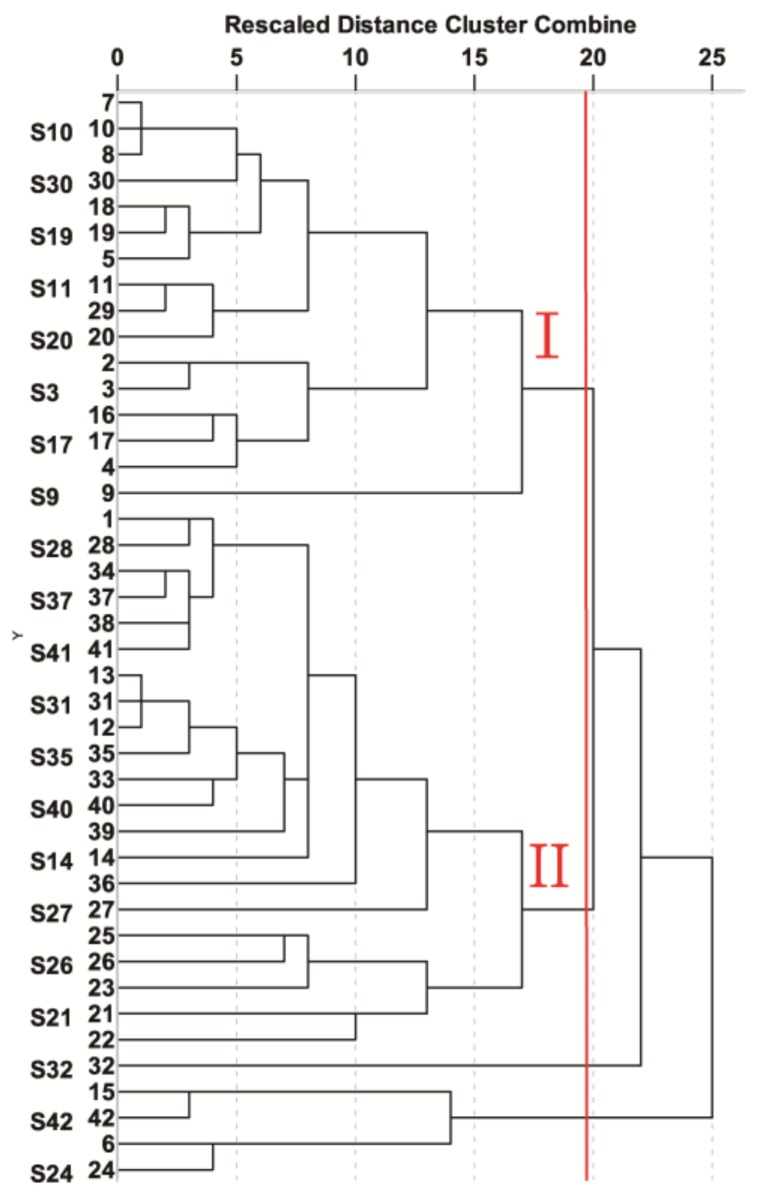
HCA of 42 bark samples of CC.

HCA provided a clear measure of similarity and variation among the different groups. HCA, which was conducted using PASW Statistics 22, showed a complex data interrelationship [25,26,27,28] among samples. Regarding hierarchical cluster analysis (HCA), a qualitative and quantitative characterization of the classification and relationships among the samples, provided a clear measure of similarity and variation among the different groups. The dendrograms are presented in Figure 4, which were constructed from 12 characteristic peaks in 42 batches of CC according to HCA based on cosine value. The dendrograms clearly show that the 42 tested samples of CC were grouped into four clusters. The compositions of the two main clusters (Cluster I: Guangdong and Cluster II: Guangxi Province) were similar to the PCA results, confirming the PCA results, indicating the rationality of both classification methods. The HCA result was consistent with the conclusions of the PCA, which indicated some geographical differences. However, compared to PCA, it is obvious that HCA can provide more classification details. 

### 2.5. Structural Characterization of the Major Active Ingredients by HPLC-DAD-ESI-MS

Many studies have shown that the quantification of the constituents of natural products in such a complex matrices is often unreliable, when they are just identified by retention time and peak characteristics with authentic reference standards. The retention time data and UV spectra are sufficient to identify the constituents in complex matrix. Owing to the complexity of compounds in natural products, accurate quantification of such complicated compounds can be a great challenge. It is well reported that due to the specific product ions from the precursor ion fragmentation [19], UPLC/MS/MS, as an effective tool, can be used for simultaneously identifying complicated compounds in herbal medicines [20]. Due to acquisition of the quasi-molecular ions and the product ions, the identification of the target analyses is more reliable. In our study, the phenylpropanoids and phenolics were firstly separated and identified by UPLC-DAD. HPLC-ESI-MS/MS was utilized to confirm the structures. The chromatographic methods of both systems used the same chromatographic conditions to ensure consistency. Identification was based on comparisons of chromatographic behavior, retention time, UV absorption, *m*/*z* of quasi-molecular ions, and MS^2^ fragmentation patterns (Table 1) with those of authentic reference substances. Table 2 shows the retention time, UV absorption, MS_1_ and MS_2_ spectral data. The structures are presented in Figure 1. The maximum absorptions of most peaks were acquired at 275–290 nm in Table 2. However, there were still difference among these compounds, such as peak **4** and peak **7** with maximum absorptions at 340 and 250 nm, which correspond to Con and Cal, respectively. The UPLC-DAD chromatograms are presented in Figure 2A, and 11 phenylpropanoids or phenolics were identified in the samples. A satisfactory chromatographic separation of phenylpropanoids and phenolics was acquired within 20 min in such a complex herbal drug system. 

### 2.6. Method Validation

#### 2.6.1. Calibration Curves, Limits of Detection and Repeatability

To evaluate the linearity, a series of suitable concentrations of standard solutions were acquired by diluting it with the standard solutions. Different concentrations of each standard solution were injected in triplicate. The peak areas were derived from the mean of each analysis and concentration. External calibration was used to quantify the analyses. The calibration curves for every standard plot the concentrations of each standard solution to the peak areas (r > 0.999). To assess the repeatability of the method, the extraction of CC was analyzed in six replicating. Table 2 lists all results.

#### 2.6.2. Precision and Stability

Precision was determined according to intra- and inter-day variation. Intra-day precision was assessed using three concentrations (low (L), moderate (M), high (H) of mixed standard solutions under the optimized conditions. Each concentration was analyzed five times within 1 day. Inter-day precision was validated with the mixed standard solutions in five replicates once a day for three consecutive days. To evaluate stability, the sample solutions were exposed at room temperature (25 °C) over two consecutive days at six time points (0, 2, 4, 6, 8, 12 and 24 h). RSD was used to express the variation. Table 2 presents all data. The results showed that the samples were stable for 24 h.

#### 2.6.3. Recovery

Three concentrations of the 11 standard analyses in CC were prepared (approximately equal to 80, 100 and 120% of the typical concentration). The method of standard addition was employed to perform recovery; the three concentrations were added to the known sample. Spiked samples were analyzed in three replicates for each concentration, as described in Section 2 and Section 3. The mean recovery were calculated using the following formula (Table 2).

### 2.7. Quantitative Application

**Figure 5:**
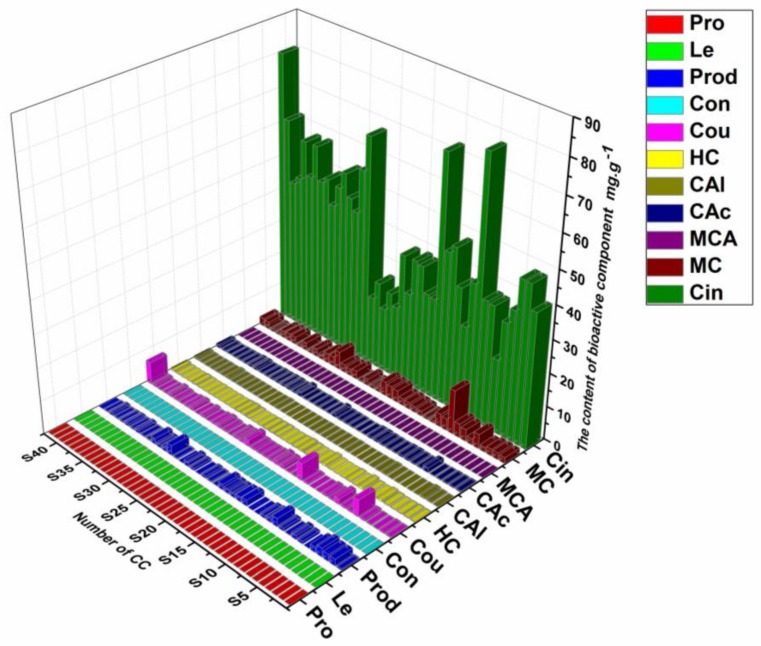
Quantitative analysis of the 11 constituents of 42 batches of CC (n = 3).

A total of 11 compounds (Figure 2A) were determined quantitatively, as a complementary quantification method of chromatographic fingerprinting. The established analytical method was successfully used to analyze 42 batches of CC samples. All 11 active compounds were determined simultaneously and the results are listed in Figure 5. In fact, there is a remarkable difference in the content of the main bioactive components. A great variation of the content of identical compounds from different samples was found, which indicate that it important for us to study this crude product. Therefore, it is also necessary that the main bioactive components of CC be quantified, especially micro active componentd, as a complement to similarity evaluation. The results of this comprehensive quantitative analysis showed that Cin is the most abundant component of the 11 bioactive components in all CC samples, ranging from 20.8 to 80.1 mg·g^−1^, almost a four-fold range. There were Cou, Cac and Mc in all CC samples, whose contents were more than 0.1 mg·g^−1^. Moreover, the contents of Mc in all CC samples were not less than 0.5 mg·g^−1^, which is deemed to be the second highest abundant component of the 11 bioactive components, ranging from 0.6 to 13.3, more than a twenty-fold range, indicating a significant difference between the samples. There were Prod, Hc and Cal, whose contents were mostly more than 0.05 mg·g^−1^, which is deemed to be the third level. Pro, Le, Con and Mca were trace compounds, whose contents were less than 0.01 mg·g^−1^. The above results confirmed there is a remarkable difference in the content of the main bioactive components of CC, especially among the micro active components. Undoubtedly, the results indicate that the quality of CC and its preparations de evaluated well with the quantification of these 11 compounds.

### 2.8. Comprehensive Evaluation of Different Cinnamomi Cortex Growth Stages 

#### 2.8.1. Hierarchical Clustering Analysis (HCA) of Different Growth Stages Cinnamomi Cortex

**Figure 6:**
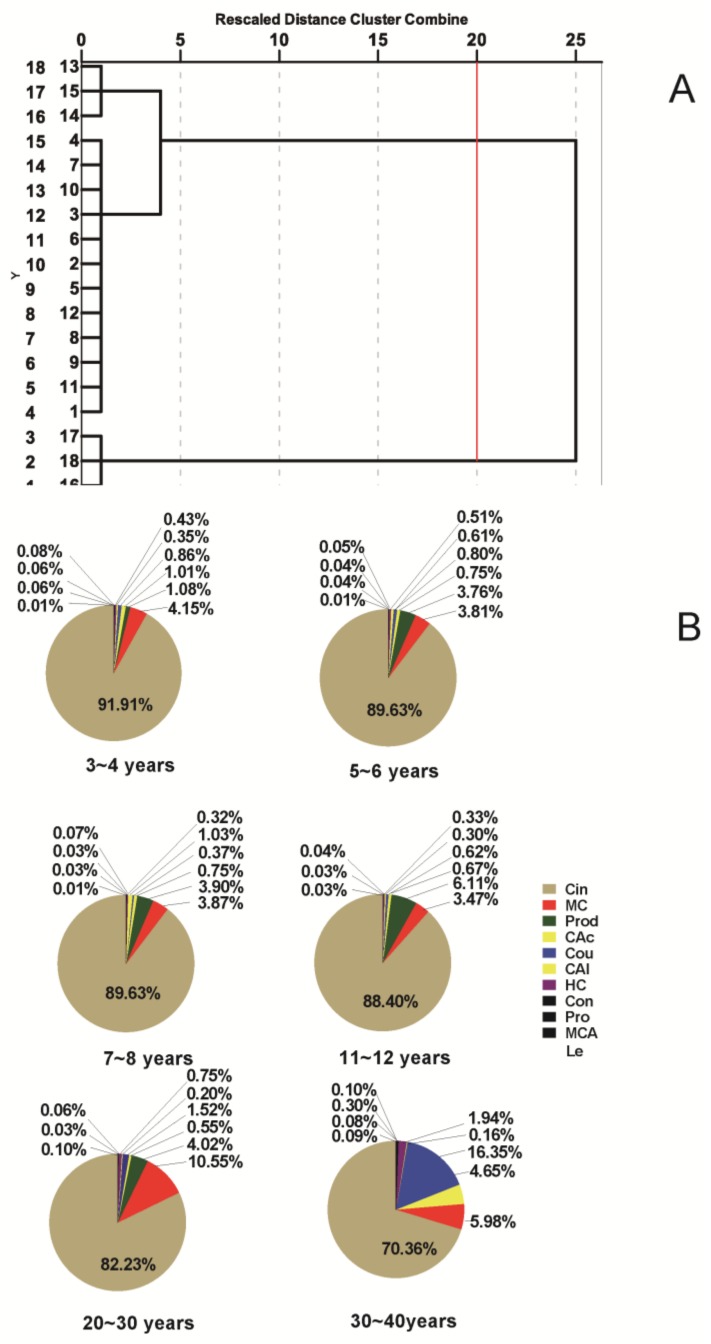
Dendrograms of HCA (**A**) of CC at different growth stages and abundance (**B**) of phenylpropanoids and phenolics in CC at different growth stages.

The dendrograms (Figure 6A) were build according to HCA based on cosine value. This revealed the relationships and distribution patterns among CC in the same place (Gaoyao County, Guangdong Province, China) at different growth stages. The areas of 12 characteristic peaks in 42 batches of CC samples were selected as the clustering variable. The dendrograms clearly suggested that the six tested samples of CC were divided into two main categories (Cluster I: S6 30–40 years; Cluster II: S5 20–30 year; S1 3–4 years; S2 5–6 years; S3 7–8 years; S4 11–12 years). The classification of results were consistent with the annual growth rings. The HCA tree showed a remarkable distinction between Cluster I and Cluster II, from which distinction can be clearly inferred between 20–30 years and 30–40 years of CC growth. However, a small distinction in the HCA tree of Cluster I, indicated that there were few differences between the 11 principal biological compounds for less than 30 year-old CC. This evidence that old growth CC is often used in the clinic, especially by famous TCM doctors in China.

#### 2.8.2. Quantitative Application of Different Cinnamomi Cortex Growth Stages

Figure 6B presents the results obtained in the determination of phenylpropanoid and phenolic compounds of CC in the same place (Gaoyao Country, Guangdong province, China) at different growth stages by the validated UPLC method. The relative abundance of components (i.e., the percentages of the 11 total components) of those samples showed some similarities as well as marked differences. In all samples, the Cin was the most abundant component, ranging from 70.36 to 91.91%. Moreover, Figure 6 shows the variation of the relative percentages of Cin among the 11 total components that was negatively correlated with the rings of CC at the same location. However, the second most abundant component differed in the six samples. Mc was the second most abundant component in the samples of 3–4, 5–6 and 20–30 years, and Prod was the second most abundant component in the samples of 7–8 and 11–12 years, whereas Cou was the second most abundant component in the samples of 30–40 years. The percentage of the other nine components also varied considerably, particularly for trace components like Prod, Con, Pro, Mca, Le.

Thanks to their similar chemical structures, UV spectral profiles and MS profiles, it has been mentioned in the previous sections that Con, Cou, Hc, Cal, Cac, Cin, Mca and Mc were classified as phenylpropanoids, so it was more meaningful that the percentages of Cin (i.e., the percentages of phenylpropanoids) were evaluated. The content and the relative abundance of Cin (i.e., the percentages of phenylpropanoids) of those samples showed some similarities as well as marked differences (Figure 6). Test results found that the content and the relative abundance of Cin of those samples exhibited some similarities of a slow rise and a significant decrease. However, the peak value of the relative abundance of Cin appeared slightly later than those of the content of Cin. It may have been caused by the conversion of Cin into other phenylpropanoids over time. 

### 2.9. Indentication of CC and Its Adulterants

Cinnamomi cortex (CC), known as Rougui in China, has been widely used as one of the most important drugs for warming the interior in Traditional Chinese Medicine (TCM) for thousands of years. According to the Pharmacopoeia of the People’s Republic of China, *Cinnamomum cassia* is the sole source of CC. However, other various species, such as *Cinnamomum tamala* and *Cinnamomum burmanni* (CB), are still being applied as CC for medicinal use in some areas of China [15,16]. The chemical components are quite different between CC and its adulterants, and they have different treatment effects. Thus, the adulteration of CC is a great threat for its clinical therapeutic efficacy and safety. Accordingly, a rapid and effective method was developed to distinguish CC from its adulterants. 

**Figure 7:**
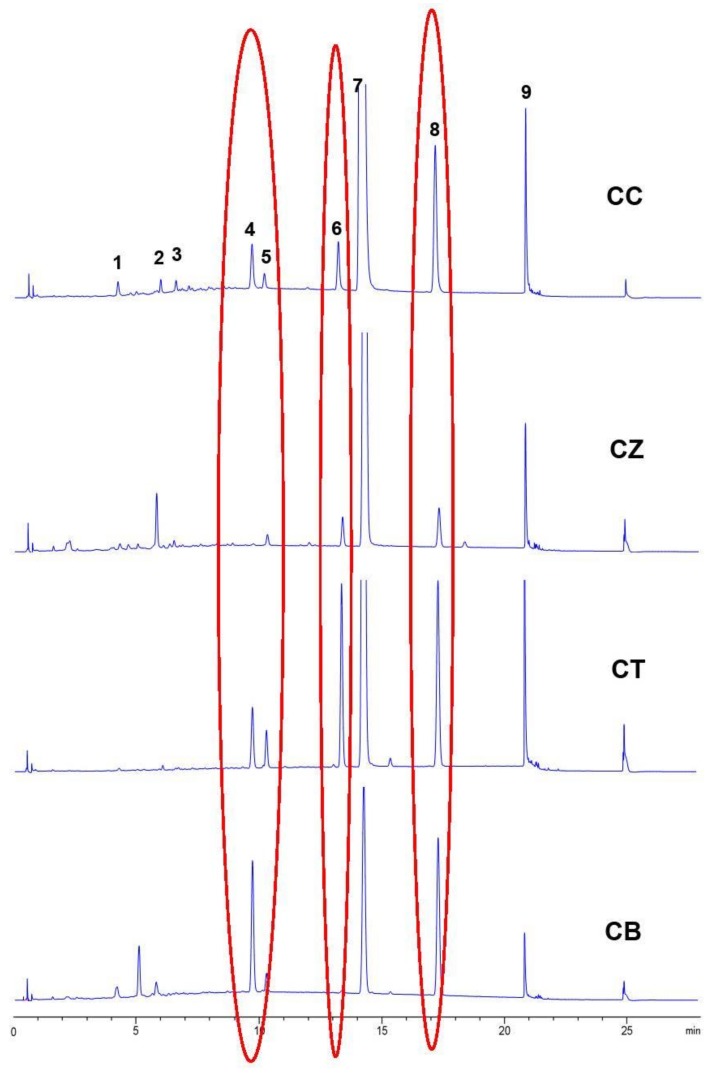
Visual classification for UPLC chromatograms of CC (Cinnamomi cortex) and its adulterants (CZ: *Cinnamomum zeylanicum*, CT: *Cassia Twig*, CB: *Cinnamomum burmanni*).

A total of 12 CC and its adulterant samples were analyzed under the optimal conditions. The representative chromatographic profile of Cinnamomi cortex (CC), *Cinnamomum zeylanicum* (CZ), *Cassia twig* (CT), *Cinnamomum burmanni* (CB) are presented in Figure 7. There were nine common chromatographic peaks, and the peaks of Cou (**4**), Hc (**5**), Cac (**6**), Cin (**7**), and Mc (**8**) were confirmed by comparing with the retention time of five reference compounds. Peak **4** (Cou), peak **5** (Hc), peak **7** (Cin) and peak were detected in all the samples, while there were some obvious differences in the relative peak areas of peaks **4** and **8**. The relative peak areas of peaks **4**, **5** and **8** in the CZ chromatograms were obviously less than in the CC chromatograms, while the relative peak areas of peaks **4**, **5** and **8** in both the CT and CB chromatograms were obviously higher than in the CC chromatogram. In addition, the relative peak area of peak **6** in the CT chromatogram was obviously higher than in the CC chromatogram, an opposite result to the CB chromatogram. The evident differences of chemical composition between CC and its adulterants came under our observation. These results can be used as the basis of why these adulterants cannot be utilized as substitutes for CC.

## 3. Materials and Methods

### 3.1. Reagents and Materials

Protocatechuate (Pro), l-epicatechin (Le), protocatechualdehyde (Prod) coniferaldehyde (Con), coumarin (Cou), cinnamyl alcohol (Cal), cinnamic acid (Cac), 2-methoxycinnamic acid (Mca) and 2-methoxycinnamaldehyde (Mc) reference substances were purchased from Shanghai Yuanye Bio-Technology Co., Ltd. (Shanghai, China), respectively. Cinnamaldehyde (Cin) reference substance was obtained from the National Institutes for Food and Drug Control (Beijing, China). 2-Hydroxycinnamaldehyde (Hc) reference substance was procured from Sigma-Aldrich (Shanghai, China). These reference standards were bought with purities ≥98%. The above reference substance meet the demands of quantitative HPLC.

HPLC-grade acetonitrile and methanol were purchased from Merck (Darmstadt, Germany). Analytical-grade formic acid was supplied from SinoPharm Chemical Reagent Ltd. (Shanghai, China). Distilled water was used throughout the study. Other solvents were of analytical grade (SinoPharm Chemical Reagent Co., Ltd., Shanghai, China).

Forty two batches of CC were purchased in February to September 2017 from genuine producing regions (Guangdong Province and Guangxi Province) and Chinese herbal medicine markets (Bozhou and Yulin). All the samples were *Cinnamomum cassia*, identified by Dr. Y. Ding (Experiment Center for Teaching and Learning, Shanghai University of TCM). Voucher specimens with the same sample numbers were deposited in Hebei Chufeng Chinese Herbal Medicine Co., Ltd. (Anguo, He’bei, China).

### 3.2. Apparatus

The fingerprinting analyses were performed with an Agilent 1290 UPLC series system (Agilent Technologies, Palo Alto, CA, USA), consisting of a quaternary pump (model G4220A), an auto sampler (model G4226A), an automatic column compartment (model G1316C), a diode array detector (DAD) (model G4214A), and a computer with Chemstation software (Agilent, Rev. C.01.07, Palo Alto, CA, USA). A TDL-50B centrifuge (Shanghai Anting Scientific Instrument Factory, Shanghai, China) was utilized for removing impurities. An ultrasonic washing unit was used for preparation of sample solutions. An electronic balance and analytical balance were employed for weighing the sample powders. Similarity evaluation system for chromatographic fingerprinting of TCM was provided from the State Food and Drug Administration of China (Beijing, China). 

### 3.3. Chromatographic Conditions

The chromatographic separation was achieved on an Agilent SB-C_18_ column (2.1 mm × 100 mm, 1.8 μm) by the chromatographic system consisted of an Agilent 1290 series UPLC with a diode array detector (DAD). After several different UPLC gradient modes were tried, a set of optimum mobile phase conditions was reached, which consisted of water solution (A) and acetonitrile solution (B) (both with 0.025% formic acid, *v*/*v*) with a gradient elution system (0–2 min, 3% B; 2–20 min 3–40% B). The temperature of column was at 40 °C. The flow-rate was set at 0.4 mL/min. The chromatographic run time for each sample was 20 min, and the injection volume was 2 uL. The wavelengths were set at 280 nm for Pro, Le, Prod, Cou, Hc, Cac, Cin, Mca and Mc; 254 nm for Cal; 340 nm for Con. The detector was set at 280 nm for acquiring chromatographic fingerprinting.

### 3.4. Preparation of Reference and Sample Solutions

Stock solutions were separately prepared by dissolving the eight concentrations in methanol:water (1:1, *v*/*v*) in order to obtain a mixed standard solution of compounds Pro, Le, Prod, Con, Cou, Hc, Cal, Cac, Cin, Mca and Mc. In order to create a series of standard working solutions with graded concentrations, the eight stock solutions were further mixed and diluted. All solutions were stored at 4 °C. The dried samples were pulverized, the powder was screened though 850 μm sieves. A 0.5 g of CC was transferred to a 25 mL conical flash with 10 mL methanol:water (1:1, *v*/*v*). The mixture was sealed, and ultrasonically extracted twice for 30 min each time. The same extract was added to the original weight, and the extracting solutions were centrifuged at 15,000 rpm for 10 min. Then the subsequent filtrate was filtered by a 0.22 μm Millipore filter, followed by injection of a 2 uL aliquot into the UPLC system. The process for ultrasonic extraction of CL and CR was similar to the process for CC, except the samples were not screened though 850 μm sieves for CL.

### 3.5. Data Analysis

The chemometrics methods including Similarity Evaluation System for Chromatographic Fingerprint of Traditional Chinese Medicine (Version 2004A, the State Food and Drug Administration of China, Beijing, China), HCA (PASW Statistics 22.0, IBM, Armonk, NY, USA) and PCA (SIMCA-13.0, Sartorius Stedim Biotech, Göttingen, Germany) were used for the evaluation of chromatographic fingerprints. 

## 4. Conclusions

The fingerprint analysis and quantity are both important components of quality control of herbal medicines and natural products. The fingerprint analysis could provide a comprehensive evaluation of the quality of CC, which could make up the restrictions of the reference and chromatographic conditions in quantification. The quantitative analysis could provide a visual comparison of variations among samples. The fingerprint analysis and quantitative support each other, combined with the chemometrics methods, which can be applied to quality control of CC. In this article, chemometrics methods including SE, PCA and HCA were successfully applied to the chromatographic fingerprints of 42 batches of CC from different areas. Twelve characteristic peaks were proved to be effective in controlling the quality of CC. Meanwhile, 11 of them were identified and determined quantitatively. The results indicated that a simple and powerful analytical method, which provided full-scale qualitative and quantitative information for evaluating the quality of CC, was established, with promising potential to play an enhanced role in the development of standards for the quality control of CC.

## Figures and Tables

**Table 1:** Identification of eight phenylpropanoids and three phenolic in Chinese Cinnamomi cortex using UPLC–DAD and HPLC–ESI–MS/MS.

Peak No.	t_R_ (min)	λ_max_ (nm)	[M + H]^+^ (*m*/*z*)	MS^2^ (*m*/*z*)	Reference
1	1.598	260 (sh), 294	155.1	137.1, 111.1, 93.2, 81.2, 65.3	Pro
2	2.590	230 (sh), 280, 310	139.2	111.1, 93.2, 65.3	Le
3	5.193	279 (sh)	291.2	207.2, 165.2, 147.2, 139.2, 123.2	Prod
4	9.092	340 (sh)	179.2	161.3, 147.2, 133.2, 119.2, 105.2, 91.2, 55.2	Con
5	9.710	279 (sh), 310	147.2	103.2, 91.2, 77.3, 65.3	Cou
6	10.312	289 (sh), 340	149.2	131.2, 121.2, 103.0, 91.2, 77.3, 55.3	Hc
7	11.987	250 (sh)	117.2	115.2, 91.2, 77.2, 65.3	Cal
8	13.353	278 (sh)	149.2	131.2, 103.2, 77.3	Cac
9	14.228	292 (sh)	133.2	115.1, 105.0, 103.1, 79.1, 77.2, 55.2	Cin
10	15.394	277 (sh), 324	179.1	161.2, 146.2, 118.2, 107.2, 103.2, 79.3, 77.3	Mca
11	17.310	288 (sh), 338	163.2	145.2, 135.2, 115.2,107.2, 105.2, 103.2, 91.2, 79.3, 77.3, 57.3, 55.3	Mc

Sh: Shoulder.

**Table 2:** Validation experimental data of 11 analytes in CC.

Analyte	Liner Range (μg·mL^−1^)	Calibration Equation	r	LOD (μg·mL^−1^)	LOQ (μg·mL^−1^)	Intra-Day RSD (%, n = 6)	Inter-Day RSD (%, n = 6)	Repeatability RSD (%, n = 6)	Recovery Mean and RSD (%, n = 6)
Pro	0.99–126.2	*Y* = 6.62*X* − 1.7136	1.0000	0.08	0.26	L 1.0	L 4.3	4.7	100.2
						M 1.0	M 1.6		
						H 0.7	H 1.2		3.3
Le	0.76–97.2	*Y* = 20.10*X* − 3.8607	1.0000	0.04	0.14	L 1.8	L 4.8	4.9	101.0
						M 0.8	M 1.6		
						H 0.5	H 1.4		2.2
Prod	0.78–99.4	*Y* = 3.23*X* − 0.8066	0.9999	0.23	0.78	L 4.0	L 2.1	3.6	100.6
						M 0.6	M 1.1		
						H 0.3	H 1.9		2.1
Con	0.97~123.6	*Y* = 33.69*X* − 7.2236	1.0000	0.04	0.12	L 0.4	L 4.0	4.3	101.0
						M 0.3	M 0.6		
						H 0.1	H 0.9		3.3
Cou	1.33–169.6	*Y* = 20.01*X* − 5.7422	1.0000	0.07	0.23	L 0.5	L 3.0	1.5	100.1
						M 0.4	M 0.8		
						H 0.2	H 0.9		3.9
Hc	0.80–101.8	*Y* = 28.842*X* − 3.5346	1.0000	0.05	0.18	L 0.3	L 4.5	1.5	101.2
						M 0.3	M 0.8		
						H 0.1	H 0.8		2.6
Cal	1.26–161.6	*Y* = 33.65*X* − 7.9961	1.0000	0.10	0.33	L 0.1	L 2.0	2.7	101.3
						M 0.3	M 0.6		
						H 0.3	H 0.9		2.8
Cac	1.61–205.6	*Y* = 31.00*X* − 28.599	0.9999	0.04	0.12	L 0.2	L 4.9	1.9	100.9
						M 0.2	M 0.7		
						H 0.3	H 0.9		3.8
Cin	2.04–260.6	*Y* = 23.31*X* − 2.5395	0.9998	0.05	0.18	L 0.8	L 1.0	0.5	98.0
						M 1.8	M 1.0		
						H 0.5	H 1.2		1.2
Mca	0.85–108.6	*Y* = 25.65*X* − 26.201	1.0000	0.08	0.25	L 0.5	L 4.4	3.9	102.6
						M 0.2	M 1.4		
						H 0.2	H 1.0		0.9
Mc	1.72–219.6	*Y*=25.56*X* − 26.201	0.9999	0.08	0.26	L 0.7	L 3.3	0.4	101.3
						M 1.3	M 0.8		
						H 0.2	H 1.0		2.8

L: low concentrations standard solutions. M: moderate concentrations standard solutions. H: high concentrations standard solutions.
